# Antibiotic Prescription Patterns in the Post-COVID-19 Era in Six European Countries: A Cross-Sectional Study

**DOI:** 10.3390/antibiotics14090911

**Published:** 2025-09-10

**Authors:** Karel Kostev, Swati Upadhyaya, Oliver Utsch, Katarzyna Sosnowska, Marcel Konrad, Christian Tanislav

**Affiliations:** 1 Philips University, University Hospital, 35043 Marburg, Germany; 2 Epidemiology, IQVIA, 60549 Frankfurt am Main, Germany; 3 Consulting, IQVIA, Bangalore 560103, India; 4 Offering Development, IQVIA, 60549 Frankfurt am Main, Germany; 5 Consulting, IQVIA, 02-672 Warsaw, Poland; 6 Health & Social, FOM University of Applied Sciences for Economics and Management, 60486 Frankfurt am Main, Germany; 7 Department of Geriatrics, Diakonie Hospital Jung Stilling Siegen, 57074 Siegen, Germany

**Keywords:** antibiotic, outpatients, Europe, post-COVID, stewardship, children

## Abstract

Background/Objective: After the relaxation of acute containment measures during the COVID-19 pandemic, Europe experienced a substantial rebound of non-COVID infections surpassing pre-pandemic levels and causing significant clinical burden. The aim of our study is to analyze outpatient prescription data in Germany, France, Italy, Belgium, the UK, and Poland in order to assess trends and disparities in the post-COVID landscape. Methods: The present cross-sectional study utilized data from six independently collected datasets containing details from longitudinal prescription (LRx) databases. We aimed to evaluate changes in the number of antibiotic prescriptions issued in 2022 (the first post-pandemic year) compared to 2021 (a pandemic year), as well as subsequent developments in 2023 and 2024. Analyses were stratified by age group and by sex. In addition, the most frequently prescribed antibiotics in each year and country were reported. Results: All countries experienced a marked increase in antibiotic use in 2022 compared to 2021. The year-on-year growth in 2022 ranged from +12.0% in France to a substantial +39.3% in Belgium. Germany, Poland, and the UK also showed strong increases of over 25%, while Italy rose by 21.5%. Growth slowed in 2023 and stabilized or declined in 2024, particularly in Poland, Italy, and Belgium. Pediatric antibiotic use surged in 2022, especially among children under 10 (+75% in the UK), then leveled off and even declined in some cases in 2024, while the number of antibiotic prescriptions in older adults either decreased or increased only slightly. Germany and the UK showed continued pediatric antibiotic use increases through 2023. Amoxicillin dominated prescriptions in most countries, but drug choice patterns varied widely by country. Conclusions: This study shows a clear increase in outpatient antibiotic prescriptions issued across Europe after the COVID-19 pandemic, particularly among children and teenagers. Although the overall trends are now starting to level out, some countries still show rising numbers. These findings underscore the importance of a renewed focus on antibiotic stewardship programs, particularly in outpatient and primary care settings.

## 1. Introduction

The COVID-19 pandemic placed a significant strain on health systems worldwide [1], although public health measures implemented to curb the spread of COVID-19 also led to a reduced incidence of previously common respiratory infections including influenza and Streptococcus pneumoniae [2,3].

After the end of the COVID-19 pandemic, however, Europe experienced a substantial rebound of non-COVID infections, particularly invasive Group A Streptococcus (iGAS) and respiratory syncytial virus (RSV)/influenza, surpassing pre-pandemic levels and causing significant clinical burden. One German review showed that after COVID restrictions were lifted, invasive Group A Streptococcus infections spiked across Europe—including Germany, Denmark, and the Netherlands—starting in fall 2022 and peaking in early 2023, with incidence rates that were notably higher than pre-pandemic levels. The surge affected children under 10 and older adults, correlating with a rebound in viral respiratory infections [4].

The resurgence of influenza and RSV, both of which had previously been suppressed under COVID-19 measures, led to atypical, off-season peaks from 2021 to 2023. For example, Denmark reported an unusually intense RSV epidemic in summer 2021, coinciding with a global relaxation of non-pharmacological interventions (NPIs) [5]. Amdor et al. reported a post-pandemic rise in the incidence and severity of pediatric iGAS in Spain, which was associated with respiratory virus seasonality and immunity gaps [6]. Lesnik et al. described an increased incidence of severe invasive GAS in adults from December 2022 to May 2023, with multiple septic shock cases and deaths in various European countries [7].

Along with the increase in infection cases, there is evidence confirming that after a pandemic-associated dip, antibiotic prescription rates have increased significantly in outpatient, community healthcare, and hospital settings, often trending above pre-2019 levels. A study using European Surveillance of Antimicrobial Consumption Network (ESAC-Net) data found that following a steep drop in 2020, antibiotic consumption began to rebound in subsequent years. This increase was significant, with sustained rises through 2022–2023 and a notable reduction in seasonal variation compared to pre-pandemic cycles [8]. Another European primary care study reported that antibiotic prescription rates climbed from 3.5% in 2022 to 4.0% in 2023, marking a relative increase of 9.5% that clearly signals a reversal of the pandemic-era decline [9].

Despite growing evidence of a resurgence in infectious diseases and rising antibiotic prescriptions across Europe in the post-COVID era, there are still significant research gaps in this area. Most of the existing studies focus on individual countries or specific pathogens and often emphasize inpatient or hospital data, with limited consideration of outpatient settings where the majority of antibiotic prescriptions are issued. Furthermore, few analyses examine how prescription patterns differ by age group or sex, despite well-established demographic variations in infection susceptibility and antibiotic use. While some studies, such as those by Vermeulen et al. [8] and Koh et al. [9], have reported general increases in antibiotic prescriptions in community practices, these do not provide stratified, comparative insights across diverse European healthcare systems or demographic subgroups. No study to date has systematically evaluated outpatient prescribing trends across multiple countries during the critical post-pandemic recovery period (2021–2024) while accounting for differences in age, sex, and national responses to COVID-19. Pre-pandemic surveillance data [10] show that outpatient antibiotic consumption in high-income European countries varied substantially, ranging from approximately 10 defined daily doses (DDD) per 1000 inhabitants per day in Germany to around 20 in France and the UK. Across countries, the overall average was ~18 DDD/1000/day during 2015–2019, before dropping sharply to ~15 DDD/1000/day in 2020–2021. These figures highlight the unusually low baseline against which post-2021 rebounds in antibiotic use must be interpreted. Previous European surveillance studies [8,10] reported that outpatient antibiotic consumption averaged ~18 defined daily doses (DDD) per 1000 inhabitants per day before the pandemic (2015–2019), dropped sharply during 2020–2021 (~15 defined daily doses per 1000 inhabitants per day (DID), and rebounded in 2022 toward pre-pandemic levels. Recent country-specific analyses, such as Waterlow et al. [11] in the UK and Koh et al. [9] in primary care, confirmed sharp post-pandemic rebounds, particularly among children. These studies, however, focus on individual countries or single healthcare sectors. To our knowledge, no prior research has systematically compared outpatient prescribing trends across multiple European healthcare systems during the critical post-pandemic recovery period.

The aim of our study is therefore to analyze outpatient prescription data in Germany, France, Italy, Belgium, the UK, and Poland in order to assess trends and disparities in the post-COVID landscape.

## 2. Results

Table 1 presents the annual number of patients receiving antibiotic prescriptions from 2021 to 2024 across six European countries. All countries experienced a marked increase in antibiotic use in 2022 compared to 2021. The year-on-year growth in 2022 ranged from +12.0% in France to a substantial +39.3% in Belgium. Germany, Poland, and the UK also showed strong increases of over 25%, while Italy rose by 21.5%.

Growth generally slowed in 2023, with most countries showing low single-digit increases. Germany remained an outlier, however, with the number of prescriptions continuing to grow significantly (+13.7%). Poland was the only country to show a slight decrease in 2023 (−2.2%), marking an early reversal.

A number of countries experienced further slight increases in 2024—France (+1.3%), Germany (+2.6%), and the UK (+4.8%)—while others saw a drop in the number of antibiotic users. Belgium (−4.5%), Italy (−1.6%), and Poland (−0.9%) recorded small declines.

Table 2 breaks down the number of antibiotic users by age group in each country from 2021 to 2024. The most prominent finding across all countries is the significant surge in antibiotic use among children and adolescents in 2022 compared to 2021. The youngest age groups (children under 10 or 13, depending on country grouping) saw increases ranging from roughly +29% in France to over +75% in the UK. Adolescents aged 10–19 (or 13–19) also showed strong rebounds, with increases exceeding +40% in several countries.

These trends then became more moderate in 2023. Antibiotic prescriptions among pediatric age groups continued to increase in some countries, especially Germany and the UK, but slowed or declined in others. For instance, France showed an increase in antibiotic prescriptions of just +4.7% in children under 10, while Italy and Poland recorded slight declines in certain age strata.

By 2024, growth had stabilized across most age groups, with numerous categories showing minimal change or small decreases. The oldest age groups (e.g., 80+) generally exhibited the least variation over time. In some cases, such as in Germany and Italy, antibiotic prescriptions in these older cohorts even declined in 2024, contrasting with continued growth in younger populations in countries like the UK.

Table 3 presents antibiotic prescription trends by sex from 2021 to 2024. All six countries experienced substantial and roughly parallel increases in antibiotic use among both women and men in 2022, with year-on-year growth typically between 20% and 40%. In most cases, increases were only slightly higher in men than in women.

In 2023, trends diverged slightly between countries. Germany and Italy saw continued double-digit growth for both sexes, while France, Belgium, and Poland experienced stabilization or modest declines. By 2024, sex-specific changes were small and largely symmetrical. Increases in the UK persisted for both sexes while France and Germany recorded further moderate growth. Italy and Belgium saw the number of antibiotic prescriptions decline or level off. Poland exhibited a contrasting pattern in 2024, with a noticeable increase in prescriptions among females (+7.0%) and a decline among males (−8.9%).

Table 4 presents antibiotic prescribing stratified jointly by age group and sex. Across all groups, prescribing increased sharply between 2021 and 2022, reflecting the rebound after the pandemic period, and then remained relatively stable in 2023–2024. In the <20 years group, boys consistently had slightly higher prescribing than girls, although the year-to-year changes were parallel. Among adults (20–59 years) and older adults (≥60 years), women had higher prescribing than men, and the relative increases between 2021 and 2022 were more pronounced in women, particularly in the ≥60 group. These patterns were broadly consistent across countries.

Table 5 reports the proportion of patients who received ≥1 prescription of each antibiotic substance per year from 2021 to 2024 across all six countries. Antibiotic distribution varied substantially between countries.

In France and Belgium, prescribing patterns were highly concentrated. Amoxicillin (including combinations with clavulanic acid) dominated antibiotic prescriptions in these countries, making up some 74–79% of all antibiotics prescribed in France and 70–73% of those prescribed in Belgium. Azithromycin was the second most common, increasing steadily over time to reach 14.7% in France and 23.6% in Belgium by 2024. The proportions of all other antibiotics declined significantly over time in both countries, making up less than 7% in France and around 6–7% in Belgium by 2024.

Germany showed more diversity in terms of antibiotic selection. Here, amoxicillin use increased from 44.0% in 2021 to 54.4% in 2024, while azithromycin use rose from 9.9% to 18.2%. Other commonly used antibiotics included cefuroxime axetil and clindamycin, although both showed a downward trend over the study period.

Italy had a similar profile, with amoxicillin use increasing steadily to nearly 60% by 2024. Azithromycin and cefixime also maintained significant shares, around 18% and 17%, respectively, in the final year. As in Germany, the proportion of less common antibiotics fell consistently over the study period.

In the UK, the pattern was relatively stable over time, with three main drugs accounting for the majority of prescriptions: amoxicillin (~51%), doxycycline (~17% by 2024), nitrofurantoin (~12%), and flucloxacillin (~11%).

In contrast to the other countries, Poland exhibited a broad and fragmented prescribing pattern. Amoxicillin, though still the most commonly prescribed drug, accounted for only 28–34% of prescriptions. Several other antibiotics—such as clarithromycin, clindamycin, azithromycin, and cefuroxime axetil—were each prescribed in moderate proportions (typically 5–9%). Notably, the “other drugs” category remained the largest or second-largest throughout, making up over one-third of prescriptions even in 2024.

To validate our findings, we compared the UK prescription-level distributions for 2023 with those reported by Waterlow et al. [11]. The proportions were very similar once nitrofurantoin was included and prescription counts were used as the denominator (Supplementary Table S2).

## 3. Discussion

This multinational analysis across six European countries highlights a clear resurgence in outpatient antibiotic prescriptions following the COVID-19 pandemic. The immediate post-pandemic period (2022) saw a substantial increase in antibiotic use, particularly among children and adolescents, followed by stabilization or moderate fluctuations in 2023 and 2024. These findings align with earlier observations of a post-pandemic rebound in infectious diseases, especially respiratory pathogens such as influenza, respiratory syncytial virus (RSV), and invasive Group A Streptococcus infections [4,5,6]. The largest year-over-year increase in antibiotic prescriptions occurred in 2022 and reflects the delayed and accumulated burden of untreated or newly emergent infections due to immunity gaps formed during prolonged lockdowns and periods of limited pathogen circulation [2]. As restrictions were lifted, these gaps likely contributed to amplified seasonal outbreaks, particularly among younger age groups whose immune systems had reduced recent exposure to common pathogens [3,6].

The age-stratified data revealed a disproportionately large increase in antibiotic use among pediatric populations across nearly all countries studied, with increases of 30–76% in 2022 alone. These findings support existing reports of unusual timing and intensity of pediatric respiratory outbreaks post-COVID [5,7].

Sex differences in prescribing were also evident. Across Europe and the UK, women are more likely than men to receive antibiotics [12] and are broadly consistent with national prescribing studies [11]. Our additional age–sex stratification shows that these differences vary by age and over time. Among individuals < 20 years, boys had slightly higher prescribing than girls, whereas from age 20 onward, women consistently received more antibiotics than men, with the largest gap seen in the ≥60 age group. Importantly, the strongest rebound between 2021 and 2022 was observed among adults and older women, suggesting that pandemic-related disruptions and recovery differentially affected age–sex groups.

Across all countries, amoxicillin (with or without clavulanic acid) remained the predominant antibiotic prescribed. Its dominance—particularly in France and Belgium, where it accounted for over 70% of prescriptions—suggests a reliance on broad-spectrum beta-lactams likely aimed at treating respiratory infections [13,14]. However, the rising proportion of azithromycin, especially in Germany and Belgium, raises concerns about macrolide overuse and potential resistance development [15,16]. Our findings are consistent with recent UK analyses by Waterlow et al. [11], who used national prescribing data to demonstrate a post-pandemic rebound in antibiotic use, particularly among children. Unlike our study, which covers six countries in parallel, Waterlow et al. [11] provide more granular UK-specific detail, underscoring the value of complementary approaches. In Germany, our analysis did not identify doxycycline among the most prescribed antibiotics, whereas Bindel and Seifert [17] reported doxycycline among the top substances. This discrepancy can be explained by methodological differences: our study is based on patient counts (≥1 prescription per year), while Bindel and Seifert report defined daily doses (DDDs) [17]. Doxycycline is often prescribed in longer treatment courses (e.g., acne or chronic respiratory conditions), resulting in a relatively small number of patients but a higher share when measured in DDDs. In contrast, clindamycin is typically used in short, high-dose courses (e.g., dental infections), producing more patients/prescriptions but fewer DDDs. This highlights the importance of distinguishing between patient-level and DDD-based metrics when comparing antibiotic use across studies. Together with ESAC-Net surveillance data [8] and primary care studies [9], these results collectively indicate that post-pandemic antibiotic prescribing rebounds are a Europe-wide phenomenon, though the magnitude and age/sex distributions differ between countries.

In Poland, the comparatively diverse and fragmented prescribing profile—with widespread use of multiple agents and a consistently large “other drugs” category—may indicate less centralized prescribing practices or differing clinical guidelines [18].

The sharp increase in antibiotic prescriptions observed in this study correlates with the documented rise in various respiratory tract infections following the COVID-19 pandemic [3,4]. However, previous research on antibiotic use has shown a high prevalence of antibiotic prescription for patients with acute respiratory tract infections of viral or undefined origin in primary care settings [19]. This suggests that the increase in antibiotic prescriptions may exceed the actual rise in bacterial infections and instead reflect the increased frequency of viral respiratory infections.

The findings of the present study underscore the importance of a renewed focus on antibiotic stewardship programs, particularly in outpatient and primary care settings, which were less prioritized in the early post-pandemic period. The exceedance of pre-pandemic antibiotic prescription levels raises alarms about potential long-term consequences for antimicrobial resistance if prescribing behavior is not aligned with appropriate clinical indications. In particular, the strong rebound in children suggests renewed and possibly excessive antibiotic prescribing as a response to uncertain clinical presentations or increased diagnostic caution, raising concerns over antimicrobial stewardship in pediatric care.

Our study has several strengths, including its use of large-scale, real-world data from six countries and consistent stratification by age and sex. However, there are also a number of limitations which have to be mentioned. A key limitation is that our LRx datasets only provided data from 2021 onwards, preventing direct comparison with pre-pandemic years. Nevertheless, published surveillance data provide important context. Simmons et al. (2021) reported that outpatient antibiotic consumption averaged ~18 DDD per 1000 inhabitants per day across several European countries during 2015–2019, with marked declines in 2020–2021 [10]. Against this backdrop, the increases we observed from 2022 onward represent both a rebound from these unusually low pandemic-era levels and, in some settings, an exceedance of pre-pandemic baselines. This context is critical when interpreting between-country differences. Moreover, the data do not include clinical indications, microbiological confirmation, or hospital prescriptions, limiting causal interpretations. Third, while robust in coverage, the IQVIA LRx datasets used do not reflect national-level prevalence of antibiotic use. Concretely, the limitation is that prescribing rates per 1000 population could not be calculated, as the LRx data are not projected to national population levels and lack population denominators. Consequently, we relied on raw patient counts and relative changes, which allow robust trend analyses but do not directly reflect absolute prescribing prevalence in the general population.

Furthermore, coverage differs between countries due to variation in pharmacy participation (30–82%), and this should be considered when interpreting absolute prescription numbers. However, because data collection and processing are consistent across countries, the observed relative year-to-year changes reliably reflect outpatient prescribing trends. A further limitation is that we could not calculate defined daily doses (DDDs), which are commonly used in European surveillance. This was not possible because the LRx data are structured at the prescription/patient level and do not consistently provide the dosing information required for conversion to DDDs. Our analysis therefore focused on trends in patient counts and proportions, which remain informative for assessing post-pandemic shifts in prescribing.

One more limitation is that our analysis was purely descriptive. Advanced statistical approaches such as interrupted time series or regression modeling would strengthen the assessment of trends, but these require patient- or pharmacy-level data that were not accessible in our aggregated dataset. Future studies with individual-level data could address this gap.

Finally, with respect to this study, data from 2021 to 2024 were available, but we did not have access to previous data (from before 2021); consequently, a comparison of pre- and post-COVID years was not possible.

## 4. Methods

### 4.1. Data Sources

This cross-sectional study utilized data from six Longitudinal Prescription (LRx) databases owned by IQVIA and updated monthly. All patient information is fully anonymized by the data provider in accordance with the data privacy laws of the respective countries (Germany, France, Italy, Belgium, the UK, and Poland). As fully de-identified datasets, the LRx databases do not require patient consent or approval from an institutional review board or ethics committee to access anonymized treatment history data.

Table 6 provides basic information on each of the six data sources used. The LRx databases in Germany [20], France, Italy, the UK, and Poland include anonymized patient identifiers, age, sex, details of the product dispensed, and prescription and dispensing dates. In Belgium, all of these variables except patient age are available (Table 1).

The coverage percentages in Table 1 indicate the approximate proportion of all prescriptions dispensed nationally that are captured in each LRx database. Coverage depends on the proportion of participating pharmacies or wholesalers and therefore varies between countries (e.g., ~30% in Belgium vs. ~82% in Germany). Importantly, there are no systematic exclusions of particular drug classes or prescriber groups. All datasets are anonymized and processed using standardized IQVIA procedures, which ensures consistency and comparability across countries. These data are widely used for pharmacoepidemiological studies and are well suited to analyzing temporal prescribing trends.

### 4.2. Outcome and Variables

The outcome of this study was the trend in antibiotic use across France, Belgium, Germany, Italy, the UK, and Poland from 2021 to 2024. We evaluated changes in the number of antibiotic users in 2022 (the first post-pandemic year) compared to 2021 (a pandemic year), as well as developments in 2023 and 2024. These trends are reported as absolute patient numbers per year and country, along with relative changes from the previous year for 2022, 2023, and 2024.

Age-stratified analyses were conducted for five countries (France, Germany, Italy, the UK, and Poland), but not for Belgium, where patient age was not available. Analyses were stratified by age group (<10 years [<13 in Poland], 10–19, 20–29, 30–39, 40–49, 50–59, 60–69, 70–79, and 80+ years [75+ in Poland]) and by sex. To examine whether sex differences varied by age, we stratified prescribing jointly by age group (<20 years, 20–59 years, and ≥60 years) stratified by sex. Information on age and sex was missing for approximately 10% of patients in each country. These patients were included in the overall analyses of antibiotic prescribing but excluded from age- and sex-stratified analyses.

Antibiotic categories were defined using ATC codes. The analysis included all systemic antibacterials (ATC J01), with individual drugs presented separately if they represented ≥5% of prescriptions in at least one country-year. All remaining antibiotics were grouped into the “other drugs” category. The full list of all antibiotic drugs used in the study is provided in Supplementary Table S1.

To facilitate comparison with previously published UK data [11] we additionally generated prescription-level distributions for the UK in 2023. While our main analyses are based on the number of patients with ≥1 prescription, this supplementary analysis used prescription counts as denominators to align with the methodology of Waterlow et al. [11] These results are presented in Supplementary Table S2.

### 4.3. Statistical Analyses

All analyses in this study were descriptive and did not involve hypothesis testing. This approach reflects the structure of the data available to us, which consisted of aggregated tabulations rather than patient-level or pharmacy-level records. While statistical modeling (e.g., interrupted time series or regression analyses) would be valuable, such analyses require individual-level data and were not feasible within the present dataset.

Given the large sample sizes, which were typically in the millions, even minimal differences (e.g., 0.1%) would result in highly significant *p*-values (e.g., <0.001). Therefore, statistical tests such as the Chi-square test were not deemed necessary. The figures presented are not extrapolated to national populations, as the study focused solely on time trends rather than estimating the prevalence of antibiotic use.

## 5. Conclusions

This study shows a clear increase in antibiotic prescription in outpatient care across Europe after the COVID-19 pandemic, particularly in children and teenagers. Although the overall trends are now starting to level out, some countries still show rising numbers, and there are differences between age and sex groups. These findings suggest that public health programs should take action to offer better guidance with regard to antibiotic prescription. Future studies should also try to connect prescription patterns with actual diagnoses and lab results to help prevent antibiotic overprescription.

## Figures and Tables

**Table 1:** Annual number of patients with ≥1 antibiotic prescription, 2021–2024.

Country *	Number of Patients with Antibiotic Prescription in Millions				Difference from Previous Year (%)		
	2021	2022	2023	2024	2022 vs. 2021	2023 vs. 2022	2024 vs. 2023
France	10.95	12.27	12.28	12.44	12.0%	0.1%	1.3%
Belgium	0.86	1.20	1.24	1.18	39.3%	3.3%	−4.5%
Germany	9.66	12.18	13.85	14.20	26.1%	13.7%	2.6%
Italy	7.12	8.65	9.72	9.57	21.5%	12.3%	−1.6%
UK	8.12	10.24	10.81	11.34	26.1%	5.6%	4.8%
Poland	11.64	15.26	14.92	14.79	31.1%	−2.2%	−0.9%

* Note: Database coverage by country is approximately France 40%, Belgium 30%, Germany 82%, Italy 70%, UK 60%, Poland 55%.

**Table 2:** Annual number of patients with ≥1 antibiotic prescription, by age group (excluding Belgium), 2021–2024.

Country * and Age Group (Years)	Number of Patients with Antibiotic Prescription in Million				Difference from Previous Year (%)		
	2021	2022	2023	2024	2022 vs. 2021	2023 vs. 2022	2024 vs. 2023
France							
<10	0.97	1.25	1.31	1.31	29.4%	4.7%	−0.2%
10–19	0.68	0.97	0.97	1.02	42.2%	0.3%	4.7%
20–29	1.24	1.32	1.28	1.27	6.6%	−3.3%	−0.7%
30–39	1.33	1.44	1.47	1.47	8.5%	2.1%	0.3%
40–49	1.38	1.50	1.51	1.54	8.5%	0.7%	1.9%
50–59	1.46	1.60	1.59	1.66	9.1%	−0.3%	4.0%
60–69	1.47	1.61	1.62	1.67	9.4%	0.6%	2.7%
70–79	1.30	1.42	1.43	1.46	9.4%	0.4%	2.1%
80+	1.11	1.15	1.09	1.05	3.5%	−5.1%	−4.0%
Germany							
<10	0.51	0.86	1.18	1.21	66.5%	37.6%	2.4%
10–19	0.39	0.70	0.94	1.09	80.9%	33.3%	16.8%
20–29	0.96	1.27	1.36	1.39	32.2%	6.8%	1.9%
30–39	1.19	1.56	1.84	1.86	31.4%	18.1%	0.9%
40–49	1.23	1.56	1.81	1.87	26.9%	16.4%	3.3%
50–59	1.36	1.66	1.85	1.95	22.3%	11.6%	5.0%
60–69	1.58	1.89	2.07	2.12	19.6%	9.7%	2.3%
70–79	1.09	1.26	1.36	1.38	15.3%	8.3%	1.3%
80+	1.35	1.43	1.43	1.34	5.5%	0.6%	−6.5%
Italy							
<10	0.43	0.71	0.99	1.03	65.1%	38.5%	4.3%
10–19	0.30	0.51	0.66	0.73	68.3%	28.8%	11.1%
20–29	0.48	0.62	0.66	0.64	28.8%	6.6%	−4.0%
30–39	0.52	0.65	0.78	0.78	24.7%	19.7%	−0.3%
40–49	0.72	0.88	1.02	1.02	21.9%	15.3%	0.0%
50–59	1.03	1.22	1.34	1.32	18.8%	9.6%	−1.2%
60–69	1.14	1.34	1.48	1.46	17.5%	10.2%	−1.7%
70–79	1.19	1.35	1.43	1.37	13.3%	6.6%	−4.3%
80+	1.30	1.36	1.36	1.22	4.8%	−0.2%	−10.0%
UK							
<10	0.70	1.22	1.31	1.43	75.9%	6.8%	9.2%
10–19	0.61	0.94	0.96	1.07	54.1%	1.7%	11.3%
20–29	0.87	1.03	1.06	1.10	18.2%	3.3%	3.7%
30–39	1.06	1.28	1.36	1.44	20.6%	6.3%	5.4%
40–49	0.96	1.15	1.22	1.29	20.2%	5.8%	5.7%
50–59	0.97	1.15	1.23	1.31	18.4%	7.2%	6.1%
60–69	1.01	1.21	1.32	1.37	20.7%	8.6%	4.1%
70–79	0.87	1.06	1.16	1.19	21.8%	8.8%	2.8%
80+	0.71	0.83	0.87	0.87	16.0%	5.4%	−0.5%
Poland							
<13	2.21	3.26	3.09	2.98	48.0%	−5.2%	−3.8%
13–19	0.57	0.81	0.85	1.05	43.0%	5.5%	22.9%
20–29	1.06	1.31	1.21	1.16	23.6%	−7.1%	−4.5%
30–39	1.57	2.05	2.00	1.81	30.7%	−2.3%	−9.5%
40–49	1.50	1.94	1.94	1.94	29.5%	0.4%	−0.1%
50–59	1.36	1.70	1.65	1.61	24.6%	−2.9%	−2.3%
60–64	0.82	0.97	0.91	0.86	18.7%	−6.4%	−5.6%
65–74	1.53	1.91	1.92	1.97	25.1%	0.4%	2.3%
75+	1.03	1.30	1.32	1.41	26.4%	1.7%	6.4%

* Note: Database coverage by country is approximately France 40%, Belgium 30%, Germany 82%, Italy 70%, UK 60%, Poland 55%.

**Table 3:** Annual number of patients with ≥1 antibiotic prescription, by sex, 2021–2024.

Country * and Sex	Number of Patients with Antibiotic Prescription in Millions				Difference from Previous Year (%)		
	2021	2022	2023	2024	2022 vs. 2021	2023 vs. 2022	2024 vs. 2023
France							
Female	6.10	6.78	6.81	6.93	11.2%	0.4%	1.7%
Male	4.85	5.49	5.47	5.51	13.0%	−0.3%	0.8%
Belgium							
Female	0.48	0.66	0.69	0.66	38.2%	3.8%	−4.1%
Male	0.38	0.54	0.55	0.53	40.7%	2.6%	−4.9%
Germany							
Female	5.45	6.84	7.75	7.91	25.5%	13.3%	2.1%
Male	4.21	5.35	6.10	6.30	27.0%	14.1%	3.1%
Italy							
Female	3.92	4.80	5.38	5.29	22.3%	12.2%	−1.7%
Male	3.20	3.86	4.34	4.28	20.5%	12.5%	−1.4%
UK							
Female	4.85	6.05	6.38	6.65	24.6%	5.4%	4.2%
Male	3.27	4.19	4.44	4.69	28.2%	5.8%	5.7%
Poland							
Female	5.99	7.69	7.50	8.03	28.4%	−2.5%	7.0%
Male	5.65	7.57	7.42	6.76	33.9%	−2.0%	−8.9%

* Note: Database coverage by country is approximately France 40%, Belgium 30%, Germany 82%, Italy 70%, UK 60%, Poland 55%.

**Table 4:** Annual number of patients with ≥1 antibiotic prescription, by sex and age, 2021–2024.

Country *, Sex, and Age Group (Years)	Number of Patients with Antibiotic Prescription in Millions				Difference from Previous Year (%)		
	2021	2022	2023	2024	2022 vs. 2021	2023 vs. 2022	2024 vs. 2023
France							
Female							
<20 years	0.81	1.07	1.12	1.15	31.8%	4.0%	3.2%
20–59 years	3.14	3.40	3.40	3.45	8.3%	0.0%	1.7%
60+ years	2.15	2.31	2.30	2.32	7.8%	−0.7%	1.1%
Male							
<20 years	0.83	1.15	1.17	1.17	37.5%	1.6%	0.6%
20–59 years	2.28	2.46	2.46	2.49	8.1%	−0.3%	1.2%
60+ years	1.74	1.87	1.85	1.85	7.6%	−1.4%	0.3%
Germany							
Female							
<20 years	0.44	0.75	1.02	1.11	68.8%	35.9%	8.8%
20–59 years	2.70	3.46	3.92	4.00	27.9%	13.3%	2.2%
60+ years	2.30	2.63	2.82	2.80	14.2%	7.0%	−0.5%
Male							
<20 years	0.46	0.81	1.10	1.20	76.4%	35.4%	8.7%
20–59 years	2.04	2.60	2.95	3.06	27.6%	13.7%	3.7%
60+ years	1.72	1.94	2.05	2.04	13.0%	5.7%	−0.7%
Italy							
Female							
<20 years	0.35	0.58	0.78	0.84	65.3%	35.0%	7.3%
20–59 years	1.57	1.96	2.21	2.19	24.8%	12.7%	−1.1%
60+ years	2.00	2.26	2.39	2.27	12.8%	5.9%	−5.2%
Male							
<20 years	0.39	0.65	0.87	0.93	67.4%	33.9%	6.7%
20–59 years	1.18	1.41	1.59	1.57	19.4%	12.2%	−1.3%
60+ years	1.63	1.80	1.89	1.79	10.1%	5.0%	−5.3%
UK							
Female							
<20 years	0.67	1.09	1.15	1.27	63.2%	5.6%	10.7%
20–59 years	2.46	2.94	3.09	3.23	19.5%	5.0%	4.4%
60+ years	1.49	1.79	1.93	1.97	19.8%	7.9%	2.1%
Male							
<20 years							
20–59 years	0.64	1.08	1.12	1.22	68.2%	3.6%	9.4%
60+ years	1.40	1.67	1.79	1.91	19.3%	7.0%	6.9%
Poland	1.10	1.32	1.42	1.46	19.9%	7.7%	3.0%
Female							
<20 years	1.38	1.93	1.88	2.25	39.6%	−2.2%	19.7%
20–59 years	2.78	3.66	3.29	3.53	31.4%	−10.1%	7.3%
60+ years	1.82	2.11	2.33	2.25	15.4%	10.6%	−3.5%
Male							
<20 years	1.39	2.15	2.06	1.77	54.3%	−3.8%	−14.2%
20–59 years	2.70	3.34	3.52	3.00	23.5%	5.6%	−14.9%
60+ years	1.56	2.08	1.83	1.99	33.9%	−12.3%	8.7%

* Note: Database coverage by country is approximately France 40%, Belgium 30%, Germany 82%, Italy 70%, UK 60%, Poland 55%.

**Table 5:** Distribution of most prescribed antibiotic substances (proportion of patients with ≥1 prescription per year) (≥5% share in at least one country per year), 2021–2024.

Country *	Antibiotic Drug	2021 (%)	2022 (%)	2023 (%)	2024 (%)
France	Amoxicillin (incl. combination with clavulanic acid)	74.0	75.3	75.7	78.6
	Azithromycin	10.2	11.5	11.6	14.7
	Other drugs	15.9	13.2	12.7	6.8
Belgium	Amoxicillin (incl. combination with clavulanic acid)	71.8	73.4	72.4	69.8
	Azithromycin	17.3	19.6	21.3	23.6
	Other drugs	10.9	7.0	6.3	6.6
Germany	Amoxicillin (incl. combination with clavulanic acid)	44.0	48.0	51.0	54.4
	Azithromycin	9.9	13.5	16.2	18.2
	Cefuroxime axetil	14.9	15.3	16.4	13.1
	Clindamycin	11.5	9.3	9.1	8.0
	Other drugs	19.7	13.9	7.2	6.4
Italy	Amoxicillin (incl. combination with clavulanic acid)	54.1	54.9	58.7	59.5
	Azithromycin	18.5	20.9	17.7	18.0
	Cefixime	13.6	15.9	17.3	17.0
	Other drugs	13.8	8.3	6.3	5.5
UK	Amoxicillin (incl. combination with clavulanic acid)	51.3	51.8	51.3	51.4
	Doxycycline	13.6	15.0	16.2	17.1
	Nitrofurantoin	13.2	11.8	11.8	11.8
	Flucloxacillin	12.5	10.8	11.1	10.8
	Other drugs	9.4	10.6	9.6	8.9
Poland	Amoxicillin (incl. combination with clavulanic acid)	28.0	32.3	33.9	30.7
	Azithromycin	9.4	10.0	8.1	8.6
	Clindamycin	7.8	7.4	6.7	8.8
	Cefuroxime axetil	6.2	6.3	6.8	5.5
	Clarithromycin	5.6	6.4	8.8	9.3
	Other drugs	42.9	37.6	35.8	37.0

* Note: Database coverage by country is approximately France 40%, Belgium 30%, Germany 82%, Italy 70%, UK 60%, Poland 55%.

**Table 6:** Basic information on the databases used in the study.

Country	Coverage (%)	Variables Included				
		Patient Identifier	Patient Age	Patient Sex	Product Details	Prescription/ Dispensing Dates
LRx Germany	~82%	x	x	x	x	x
LRx France	~40%	x	x	x	x	x
LRx Belgium	~30%	x		x	x	x
LRx Italy	~70%	x	x	x	x	x
LRx UK	~60%	x	x	x	x	x
LRx Poland	~55%	x	x	x	x	x

## Data Availability

Data were obtained from IQVIA and are available upon reasonable request with the permission of IQVIA. Restrictions apply due to data protection requirements.

## Supplementary Materials

The following supporting information can be downloaded at https://www.mdpi.com/article/10.3390/antibiotics14090911/s1, Table S1: Antibiotics included in the study and corresponding ATC codes (WHO ATC/DDD Index 2025); Table S2: Comparison of antibiotic prescription shares in the UK in 2023 between the present study and Waterlow et al. (2025) [11].
