# Supplementary material for: Antibiotic Prescription Patterns in the Post-COVID-19 Era in Six European Countries: A Cross-Sectional Study
Source: Antibiotics (Basel). 2025 Sep 10;14(9):911. doi: 10.3390/antibiotics14090911 (PMC12466364; doi:10.3390/antibiotics14090911)
Supplement: Supplementary file 1 [file antibiotics-14-00911-s001.zip › antibiotics-3818788-supplementary.pdf]

Table S1. Antibiotics included in the study and corresponding ATC codes (WHO ATC/DDD Index 2025).

**Antibiotic**	**ATC code**
Amikacin	J01GB06
Amoxicillin	J01CA04
Amoxicillin + Clavulanic acid	J01CR02
Amoxicillin + Flucloxacillin	J01CR50
Ampicillin	J01CA01
Ampicillin + Flucloxacillin	J01CR50
Ampicillin + Sulbactam	J01CR01
Azithromycin	J01FA10
Aztreonam	J01DF01
Bacampicillin	J01CA06
Cefaclor	J01DC04
Cefadroxil	J01DB05
Cefalexin	J01DB01
Cefalotin	J01DB02
Cefazolin	J01DB04
Cefditoren pivoxil	J01DD16
Cefepime	J01DE01
Cefiderocol	J01DI12
Cefixime	J01DD08
Cefodizime	J01DD09
Cefotaxime	J01DD01
Cefotiam hexetil	J01DC06
Cefoxitin	J01DC01
Cefpodoxime proxetil	J01DD13
Cefprozil	J01DC10
Cefradine	J01DB09
Ceftaroline fosamil	J01DI02
Ceftazidime	J01DD02
Ceftazidime + Avibactam	J01DD52
Ceftibuten	J01DD14
Ceftobiprole medocaril	J01DI01
Ceftobiprole medocaril	J01DI01
Ceftolozane + Tazobactam	J01DI54
Ceftriaxone	J01DD04
Cefuroxime	J01DC01
Cefuroxime axetil	J01DC02
Chloramphenicol	J01BA01
Ciprofloxacin	J01MA02
Clarithromycin	J01FA09

Clindamycin	J01FF01
Clofoctol	R02AB01
Cloxacillin	J01CF02
Colistin (Polymyxin E)	J01XB01
Dalbavancin	J01XA04
Daptomycin	J01XX09
Demecyclocycline	J01AA01
Doxycycline	J01AA02
Ertapenem	J01DH03
Erythromycin	J01FA01
Flucloxacillin	J01CF05
Flumequine	J01MB06
Fosfomycin	J01XX01
Fosfomycin / Fosfomycin trometamol	J01XX01
Fosfomycin trometamol	J01XX01
Fusidic acid	J01XC01
Gentamicin	J01GB03
Gentamicin + Collagen	J01RA10
Imipenem + Cilastatin	J01DH51
Josamycin	J01FA07
Levofloxacin	J01MA12
Lincomycin	J01FF02
Linezolid	J01XX08
Lomefloxacin	J01MA07
Lymecycline	J01AA04
Meclocycline	J01AA03
Meropenem	J01DH02
Metacycline	J01AA05
Metronidazole	J01XD01
Metronidazole + Spiramycin	J01RA04
Midecamycin	J01FA03
Minocycline	J01AA08
Moxifloxacin	J01MA14
Neomycin	J01GB05
Netilmicin	J01GB07
Nitrofurantoin	J01XE01
Norfloxacin	J01MA06
Ofloxacin	J01MA01
Oxacillin	J01CF04
Oxytetracycline	J01AA06
Penicillin G (benzylpenicillin)	J01CE01
Penicillin G + Tolycaine	J01RA01

Penicillin V (phenoxymethylpenicillin)	J01CE02
Pheneticillin	J01CE05
Piperacillin	J01CA12
Piperacillin + Tazobactam	J01CR05
Pivmecillinam	J01CA08
Pristinamycin	J01FG01
Prulifloxacin	J01MA17
Rifabutin	J04AB04
Rifampicin	J04AB02
Rifamycin	J04AB01
Roxithromycin	J01FA06
Rufloxacin	J01MA18
Spectinomycin	J01XX04
Spiramycin	J01FA02
Sulbactam	J01XX09
Sulfamethizole	J01EB02
Sulfamethoxazole + Trimethoprim	J01EE01
Sultamicillin	J01CR04
Tedizolid	J01XX11
Teicoplanin	J01XA02
Telithromycin	J01FA15
Temocillin	J01DI02
Tetracycline	J01AA07
Thiamphenicol	J01BA02
Tigecycline	J01AA12
Tobramycin	J01GB01
Trimethoprim	J01EA01
Vancomycin	J01XA01

Table S2. Comparison of antibiotic prescription shares in the UK in 2023 between the present study and Waterlow et al. (2025)

Antibiotic name	Precriptions in England in 2023 (Waterlow et al. (2025).		Prescriptions in the UK in 2023 (present study)	
	N	%	N	%
Amoxicillin	7,311,226	24.4	7,603,213	27.8

Nitrofurantoin	3,808,622	12.7	3,135,005	11.5
Flucloxacillin sodium	3,384,802	11.3	2,957,049	10.8
Doxycycline hyclate	3,804,564	12.7	3,183,192	11.7
Penicillin V	2,447,555	8.2	2,267,921	8.3
Trimethoprim	1,428,569	4.8	1,353,904	5.0
Clarithromycin	1,743,846	5.8	1,490,175	5.5
Co-amoxiclavd	1,092,620	3.7	1,064,684	3.9
Lymecycline	861,423	2.9	672,729	2.5
Cefalexin	816,579	2.7	658,857	2.4
Azithromycin	805,700	2.7	608,114	2.2
Metronidazole	474,629	1.6	461,322	1.7
Ciprofloxacin	355,804	1.2	325,158	1.2
Erythromycin	286,163	1.0	425,362	1.6
Oxytetracycline	178,452	0.6	131,462	0.5
Pivmecillinam hydrochloride	301,447	1.0	229,425	0.8
Erythromycin ethylsuccinate	164,552	0.5	124,838	0.5
Co-trimoxazolee	228,051	0.8	192,625	0.7
Methenamine hippurate	319,013	1.1	302,126	1.1
Rifaximin	109,205	0.4	96,063	0.4
Rest	unknown		32,040	0.1
Total	29,922,822		27,315,264	
